# New Advances in Metabolic Syndrome

**DOI:** 10.3390/ijms25158311

**Published:** 2024-07-30

**Authors:** Manfredi Rizzo, Ali A. Rizvi

**Affiliations:** 1 School of Medicine, Promise Department, University of Palermo, 90100 Palermo, Italy; 2 Department of Medicine, Division of Endocrinology, Orlando VA Medical Center and University of Central Florida College of Medicine, Orlando, FL 32827, USA

It is with great anticipation and pride that we present a Special Issue entitled “New Advances in Metabolic Syndrome”, which provides a compendium of high-quality original papers written on novel aspects of metabolic syndrome (MetS). These novel publications have been authored by prominent leaders in the field. As a quick reminder to the reader, the MetS is the name given to a cluster of derangements that encompass insulin resistance, central obesity, hyperglycemia, hypertension, and lipid abnormalities. Two key aspects of this condition render it especially lethal—namely, the vastly increased risk of developing type 2 diabetes and cardiovascular disease and the alarming rapidity that it is increasing in prevalence [1]. The latter is inextricably intertwined with the burgeoning obesity epidemic worldwide. Public health measures targeting early identification and prevention are key to reversing this trend. Recognizing clinical indicators and relevant biomarkers for diagnosis is essential to mitigate the impact of the MetS in our communities. However, developing a basic understanding of the pathways that underlie the MetS and play a central role in its evolution is equally important in halting adverse impacts on human health. Genetic breakthroughs and molecular insights are already underway in this respect. Equally significant is knowledge of the etiologic and pathophysiologic pathways that could facilitate novel therapeutic approaches to managing the manifestations of the MetS [2]. This Special Issue encompasses a variety of peer-reviewed articles that are diverse in their range of research while exploring new aspects of the condition. 

A total of seven original papers in this Special Issue advance our knowledge of genetic susceptibility, obesity features, and novel management approaches to obesity, metabolic syndrome, and hyperglycemia in both animal and human studies. Glass et al. and Udosen et al. investigate blood pressure findings in special populations. This topic is a particularly relevant target of intense research [3]. The former found that the debilitating condition of myalgic encephalomyelitis/chronic fatigue syndrome (ME/CFS) is characterized by a relative lack of adaptation to stress, as evidenced by urine metabolomic studies. The latter meta-analysis of over 80,000 African subjects identified certain novel genetic variants identified with traits leading to increased susceptibility to elevated blood pressure. Glucose disturbances were noted by Bergmann and associates to signify a tendency for prediabetes in the pediatric population, while Khvostov’s group detailed the glucose-lowering properties of novel 9-N-heptyltetrahydroberberine derivatives in mice that exhibited features of MetS. The development of hepatocellular carcinoma in a mouse model of steotohepatitis has ominous overtones when extrapolated to individuals prone to fatty liver and MetS, as shown by O’Neill and others in yet another high-quality paper in this issue. In the paper by Shao et al., obese mouse models of steatohepatitis were prone to developing hepatocellular adenomas, of which the majority expressed glutamine synthetase, which is a marker of hepatocellular carcinoma. This brings home the connection between obesity, liver dysfunction, and neoplastic risk. Finally, Hidalgo-Bravo and colleagues elegantly explore a genetic predisposition to increased cardiovascular risk by reporting the frequency of two commonly occurring single nucleotide polymorphisms in Mexican subjects with metabolic syndrome.

The three reviews incorporated in this Special Issue are striking in their timeliness and diversity. The emerging importance of the role the gut microbiome plays in health and disease is expertly condensed by Bondy, while Ionescu and colleagues also provide a comprehensive overview of this in patients with gestational diabetes. Finally, an almost inescapable connection between MetS and Alzheimer’s disease is reinforced by Ezkurdia and coauthors; more importantly, however, the focus on insulin resistance as an underlying pathophysiologic mechanism is emphasized by the authors, which is a notion that is gathering momentum in the medical literature [4]. An emerging and more complete picture of the pathophysiology of the metabolic syndrome [5] is shown in Figure 1, which is borrowed from an open-access publication by the same authors.

In conclusion, this Special Issue aims to provide an update on the latest research in MetS, shedding light on emerging markers, unraveling molecular mechanisms, and providing innovative remedies. The link between MetS and glucose intolerance, diabetic complications, and atherosclerotic disorders is a rapidly evolving archetype. Accumulating evidence points to the confluence of underlying genetic predisposition, heightened endovascular inflammation, and metabolic derangements as unifying abnormalities. It is our hope that the combination of original investigative reports and timely reviews of current knowledge included in this issue will add to the rapidly growing information from which future therapeutic breakthroughs might emerge.

## Figures and Tables

**Figure 1:**
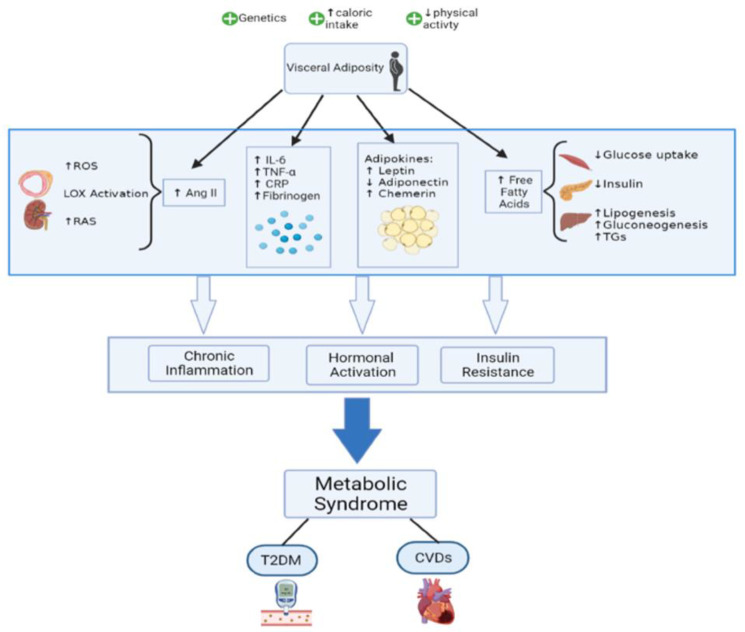
Mechanisms highlighting the pathophysiology of the metabolic syndrome (from ref. [5]: Fahed et al. Metabolic Syndrome: Updates on Pathophysiology and Management in 2021. *Int. J. Mol. Sci.* **2022**, *23*, 786. Open access, available at https://doi.org/10.3390/ijms23020786).
